# Coexistence of Kaposi sarcoma and Molluscum contagiosum on the same site in a HIV-AIDS patient: A very rare occurrence

**DOI:** 10.4102/ajlm.v8i1.747

**Published:** 2019-04-29

**Authors:** Kabir Abdullahi, Yahaya Mohammed, Saddiku A. Sahabi, Mahmood M. Dalhat

**Affiliations:** 1 Department of Morbid Anatomy and Forensic Medicine, Faculty of Basic Medical Sciences, College of Health Sciences, Usmanu Danfodiyo University, Sokoto, Nigeria; 2 Department of Medical Microbiology and Parasitology, Faculty of Basic Medical Sciences, College of Health Sciences, Usmanu Danfodiyo University, Sokoto, Nigeria; 3 Nigerian Field Epidemiology and Laboratory Training Program, Abuja, Nigeria

**Keywords:** Kaposi sarcoma, Molluscum contagiosum, HIV, AIDS

## Abstract

**Introduction:**

There have been numerous reported opportunistic infections among HIV/AIDS patients. However, coexistence of Kaposi sarcoma and Molluscum contagiosum on the same site is a rare finding.

**Case presentation:**

A 37-year-old man poorly adherent to antiretroviral therapy presented with Molluscum contagiosum and Kaposi sarcoma occurring simultaneously on numerous skin lesions around mid-2017 at Usmanu Danfodiyo University Teaching Hospital, Sokoto State, Nigeria.

**Management and outcome:**

The patient was counselled and re-initiated on a second-line highly active antiretroviral therapy regimen. The patient’s lesions resolved three months later.

**Discussion:**

The case is presented to improve the index of suspicion among clinicians and pathologists on such rare occurrences.

## Introduction

Molluscum contagiosum has been classified as an AIDS-defining illness; it usually causes a self-limiting skin lesion, but can become widely disseminated. It has a predilection for the head and neck area of individuals with AIDS.^1^ Lesions range in size from 0.2 cm to 0.6 cm, although giant forms have been reported. They classically have an umbilication. On the other hand, Kaposi sarcoma has a more generalised distribution, affecting more organs and systems. The lesions of Kaposi sarcoma are usually purple in colour, flattened or raised and they are more difficult to manage and contribute more to mortality.^1,2^

Coexistence of Kaposi sarcoma and Molluscum contagiosum in the same patient is rare and more difficult to diagnose and manage. We present a case report of a patient living with HIV, with multiple skin lesions, for which both diseases were diagnosed.

## Ethical considerations

Ethical approval to conduct the study was sought and obtained from the Health, Research and Ethics Committee (HREC) of Usmanu Danfodiyo University Teaching Hospital, Sokoto, Nigeria, with approval number UDUTH/HREC/2018/No. 658. Consent and permission were obtained from the patient to use his picture and details for the study.

## Case presentation

**FIGURE 1:**
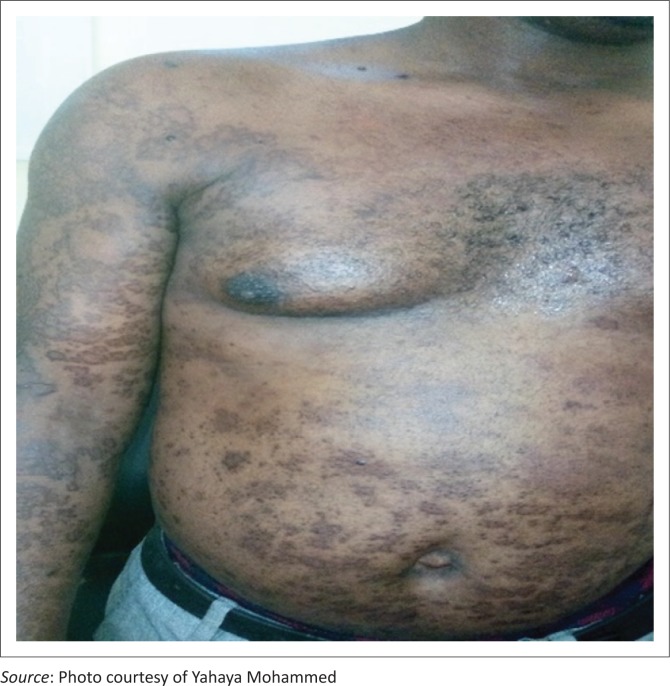
Anterior view of the skin lesion diagnosed as Kaposi sarcoma and Molluscum contagiosum.

**FIGURE 2:**
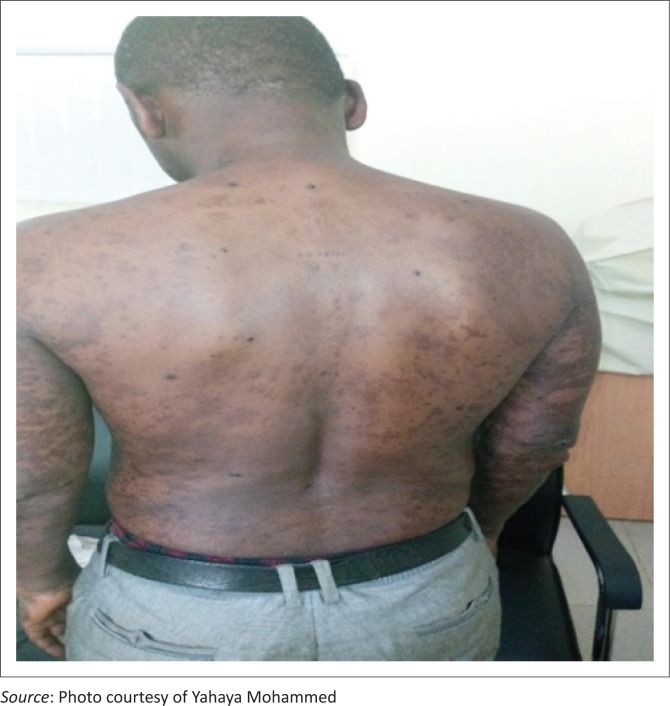
Posterior view of the skin lesion diagnosed as Kaposi sarcoma and Molluscum contagiosum.

A 37-year-old man who had been HIV-positive for 2 years later became poorly adherent to first-line antiretrovirals (defaulted for more than 6 months). He re-presented at our facility again with low CD4 count (98 cells per *µ*L) and high viral load (> 10 000.00 copies/mL) and had developed progressive, generalized, asymmetrical, non-scaly, maculo-papular, hyperpigmented focally nodular cutaneous lesions involving the head, neck, trunk (anteriorly and posteriorly), and the proximal upper and lower limbs, especially on the medial surfaces with the largest nodule reaching 2.5 cm in diameter, over a period of 3 months (Figure 1 and Figure 2). He was on a first-line highly active antiretroviral therapy regimen (zidovudine/lamivudine/nevirapine) before defaulting. We conducted a skin biopsy for histopathology.

### Histopathological findings

**FIGURE 3:**
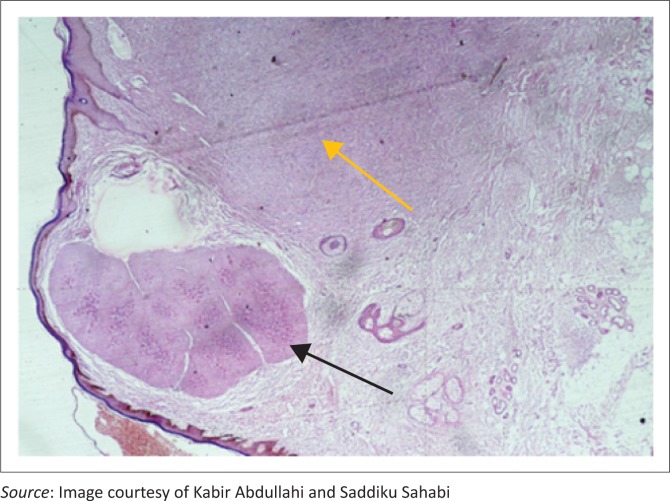
Low power view of the coexisting Kaposi sarcoma (golden arrow) and Molluscum contagiosum (black arrow). Haematoxylin and eosin staining X 40.

**FIGURE 4:**
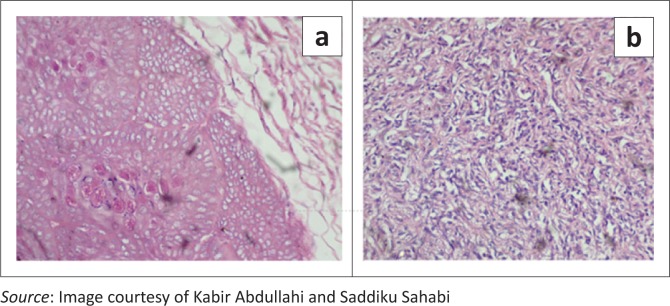
(a) Section showing a lobular lesion composed of enlarged keratinocytes with central eosinophilic molluscum bodies. (b) Section showing plump spindle cells with bland nuclei delimiting slit-like vascular spaces, consistent with Kaposi sarcoma. Haematoxylin and eosin staining X 200.

The laboratory received a small tissue fragment measuring 3 cm × 2 cm × 2 cm fixed in 10% buffered formalin. Tissue was sectioned following processing and embedding in paraffin wax. Light microscopy conducted on the haematoxylin and eosin stained tissue revealed a cellular nodular tumor composed of slit and sieve-like spaces containing red blood cells. These spaces were lined by plump dark cells with eosinophilic cytoplasm. In another focus within the lesion was a lobular lesion composed of enlarged keratinocytes whose nuclei were distended by eosinophilic amorphous bodies, consistent with molluscum bodies (Figure 3 and Figure 4). These findings are pathognomonic of both Kaposi sarcoma and Molluscum contagiosum (coexisting).

## Management and outcomes

The patient was counselled and re-initiated on a second-line highly active antiretroviral therapy regimen (tenofovir/lamivudine/lopinavir). He was re-evaluated three months after re-initiation. He has since been compliant (current CD4 count of 450 cells per *µ*L and an undetectable viral load of < 20 copies/mL). The patient’s lesions resolved, even though no dermatological procedures or creams were used.

## Discussion

Kaposi sarcoma and Molluscum contagiosum both have viral infectious aetiologies, and commonly occur when the CD4 cell count is less than 150 cells per *µ*L.^2^ The former is caused by a herpes virus, and the latter by a pox virus.^2^ Our patient most likely had the infection either as reactivation or new infection during his period of non-adherence when his CD4 count and viral load deteriorated. We initially had a clinical suspicion of lepromatous leprosy due to the widespread nature of the lesions but the absence of nerve involvement and loss of sensation ruled it out.

The hallmark of AIDS is increased susceptibility to opportunistic infections.^3^ Kaposi sarcoma and Molluscum contagiosum are categorized as AIDS-defining illnesses. Even though their coexistence^4^ in HIV/AIDS patients has been widely described, the occurrence of the two diseases side by side within the same lesion is a rare occurrence.^5^

This case highlights why it is necessary to have a high index of suspicion when dealing with immunocompromised patients from clinical examination to sampling, during biopsy and ultimately in interpretation for the coexistence of skin diseases.

### Conclusion

Clinicians and pathologists should be mindful of unusual presentations of opportunistic AIDS-defining illnesses in HIV/AIDS patients. Our index patient was poorly adherent to treatment resulting in low CD4 counts and high viral loads. Consequently, all efforts should be made to ensure adherence to treatment by HIV patients to optimise outcomes. The case report also highlights the importance of laboratory investigations and the evidence they provide in making accurate diagnoses in a patient population that is known to be at risk for multiple opportunistic infections affecting the same organ at the same time. We present this case, because of its unusual occurrence and also the need to counsel patients on compliance to highly active antiretroviral therapy medication once diagnosed with HIV/AIDS.
